# Investigations on the Deflection of Carbon-Reinforced Concrete Hollow-Core Slabs

**DOI:** 10.3390/ma18061212

**Published:** 2025-03-08

**Authors:** David Sandmann, Michael Frenzel, Steffen Marx, Manfred Curbach

**Affiliations:** 1 Institute of Concrete Structures, TUD Dresden University of Technology, 01062 Dresden, Germany; steffen.marx1@tu-dresden.de (S.M.); manfred.curbach@tu-dresden.de (M.C.); 2 Kahnt & Tietze GmbH, 04317 Leipzig, Germany; michael.frenzel@kahnttietze.de

**Keywords:** carbon-reinforced concrete, CRC, analysis, deflection, slab test, analytical calculation, carbon grid, hollow-core slab, bending, house, ceiling

## Abstract

The article presents the experimental and computational investigations on carbon-reinforced concrete (CRC) slabs with hollow-core cross-sections. Designed for use in building construction, they combine the benefits of lightweight construction, resource efficiency, and precise prefabrication. Three geometrically identical elements were manufactured and tested until failure in four-point bending tests. The slabs demonstrated a high load capacity of around 50 kNm, together with high ductility due to a deformation of more than 80 mm before failure. The load-deflection curves recorded could be reproduced very well with the analytical-physical calculation model created for both the non-cracked and cracked slab states. The strengths and stiffnesses of the materials used for input were derived from small-scale, accompanying material tests. As a result, the calculation model was ultimately used to design the carbon-reinforced ceilings of the CRC technology demonstration house CUBE, which was finished in 2022 in Dresden, East Germany.

## 1. Introduction

The construction of the world’s first carbon-reinforced concrete (CRC) house “CUBE” was finished in Dresden, East Germany, in September 2022. It is a building in which the concrete was reinforced exclusively with non-metallic reinforcement. The main materials used were technical grids made of impregnated carbon yarns and profiled rebars made of carbon and glass fiber composites. These are characterized by high tensile strength and durability and can be installed in the concrete with a comparatively small concrete cover. They contribute to the realization of slender, sustainable, and visually appealing concrete structures. This has already been shown in various applications such as slabs [1] or bridges [2]. Especially for the slabs, studies show the transfer of the advanced properties of the carbon reinforcement compared to steel into high load-bearing capacities together with significant reductions in the dead load and the materials for the concrete. In contrast to the large deformations that are desired when the load-bearing capacity is reached, small deformations are required under normal service loads in order to achieve high ductility and sufficient early indication of failure. Therefore, in a re-introduced, efficient design approach, a hollow-core section was considered, which has comparable material savings to a thin solid section, but limits the deformations due to a large internal lever arm and, hence, moment of inertia. With this in mind, and in contrast to the previous studies, the deformation behavior of the elements under investigation and its prediction was of particular interest.

**Figure 1:**
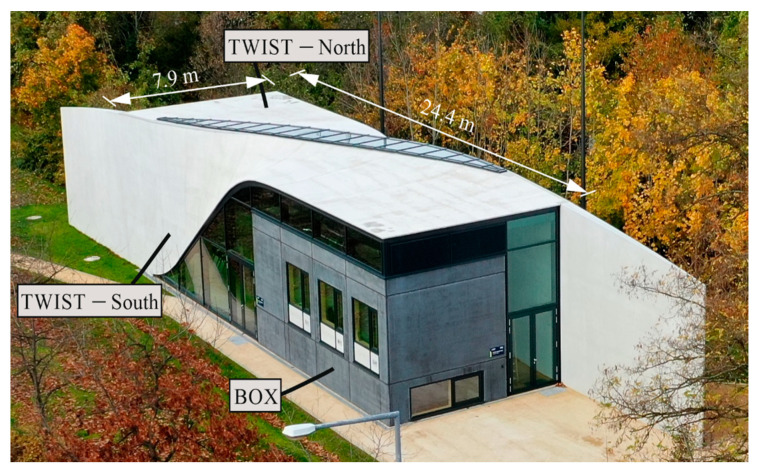
C^3^ technology demonstration house CUBE [8]. Photo: Gröschel (IMB TUD).

The CUBE building was constructed at the end of the major construction research project “C^3^—Carbon Concrete Composite” [3] (duration: 2014–2022) and represents its results and those of previous carbon-reinforced concrete research projects. In addition, the CUBE team showed that the entire process to realize a fully functional building made of carbon-reinforced concrete works. This includes planning, design, tendering, construction, and operation of a building [4,5]. The building measures a footprint of approximately 24.4 m × 7.9 m = 193 m^2^ and a height of up to 7.0 m. It consists essentially of three parts: the two-story BOX made of prefabricated elements, two twisted, geometrically identical TWIST shells arranged offset to each other, and a steel glass façade (Figure 1). The two TWIST shells extend beyond the building-like wings, giving it a total length of 40.1 m. The shells consist of a slender multi-layered structure of CRC in situ shotcrete, insulation, and sealing. Detailed information can be found in [5]. While the shells demonstrate the suitability of CRC for the production of curved, organically shaped surfaces, the BOX demonstrates its suitability for the economical production of mass-produced cuboidal components such as walls and ceilings. The detailed design is presented in [4]. The building serves as a research structure for observing the long-term behavior of carbon-reinforced concrete on a real scale.

As a basis for the calculation and dimensioning of the installed carbon-reinforced hollow core precast ceiling slabs, tests on beam-like strips on a scale of 1:1 were carried out at the Otto-Mohr Laboratory (OML) of the TUD Dresden University of Technology (see also [6,7]). This paper presents the large-scale component tests performed, including the small-scale specimens, the test procedure, and the evaluated results. In addition, the verification of the calculation models for the determination of the ultimate load and the component deflections in the cracked and uncracked states are emphasized. Previous studies were strongly focused on the ultimate limit state in terms of load-bearing capacity. However, since service conditions play a special role in more slender CRC structures, the present study addresses this issue. On the one hand, it was shown that high stiffness against deformation can be achieved with a small, intelligently arranged cross-sectional area. On the other hand, the deformation behavior of CRC components was recalculated using practical manual calculations and the usual deformation principles of reinforced concrete construction. This means that not only can tests be successfully recalculated, but also new components can be designed.

## 2. Materials and Methods

### 2.1. Materials

#### 2.1.1. Carbon Reinforcement

**Figure 2:**
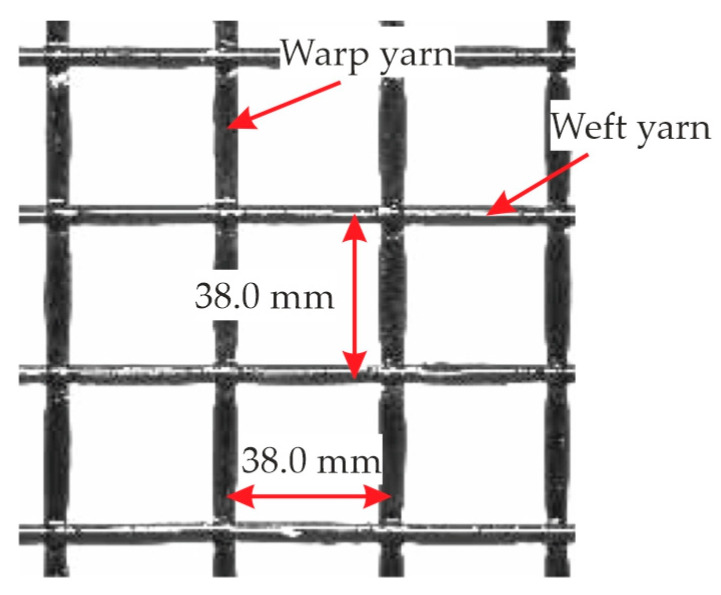
Biaxial carbon textile grid Q95/95-CCE-38-E2. Photo: Scheerer (IMB TUD).

For the planar slab elements of the CUBE building, biaxial carbon textile grids were used. These were provided by solidian GmbH (Albstadt, Germany) called solidian Q95/95-CCE-38-E2 (briefly: Q95) [9]. Each yarn of the grid consists of 96,000 (96 K) carbon filaments resulting in a fiber cross-sectional area of the fiber strand *A*_f,nm_ of 3.62 mm^2^. The spacing of the yarns is 38 mm, as shown in Figure 2. This gives a total cross-sectional reinforcement area of 95.3 mm^2^/m. The impregnation material is epoxy resin, which ensures a good internal bond between the filaments as well as an external bond to the concrete. The geometric material properties of Q95 are summarized in Table 1. Further details are given in [10].

The bond behavior of the yarns to the concrete is highly influenced by the fiber and impregnation materials, the geometry, and the surface properties. The surface of the selected carbon grid is smooth. The bond strength can be increased by sand-coating the grid as shown in [11] or by varying cross-sectional thickness along the length of the yarn to achieve a small amount of mechanical interlock similar to steel reinforcement, e.g., [12].

The mechanical material properties and test results of the reinforcement are discussed in Section 2.2.2.

#### 2.1.2. Concrete

**Figure 3:**
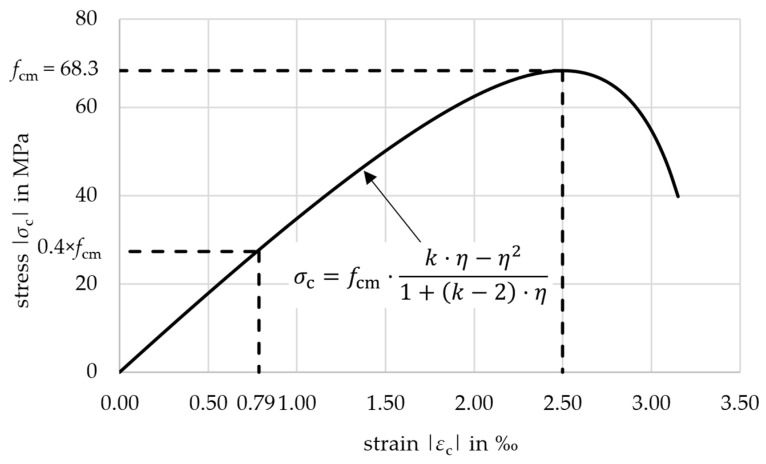
Stress–strain relationship of the concrete.

The concrete was specially designed for use with non-metallic carbon reinforcement. It has a maximum grain size of 8.0 mm and a high content of fine particles and cement paste, which is necessary for good bonding to the reinforcement. The used cement type was CEM I 52.5 N. The mixture with its components is listed in detail in [10]. It was originally designed for classification in a strength class C50/60 according to [13]. This was especially necessary for the fast removal of the formwork in the economic and efficient production process of the prefabrication factory. This requires a high early strength after 24 h. To determine the actual material properties of the concrete, several tests on different specimens were conducted. Concrete cubes with an edge length of 150 mm were cast, stored in water, and tested according to [14,15,16] to determine the concrete strength class prior to the large-scale component tests. They gave a compressive strength of *f*_cm,cube_ = 73.8 MPa after 28 days, which was a little higher than planned. The concrete mixture was therefore classified in the strength class C55/67. To recalculate the large-scale component tests, results from cylindric specimens (height/diameter = 300/150 mm) were used. The cylinders were concreted in the same batch as the components and stored under the same conditions. Values for Young’s modulus, compressive strength, and splitting tensile strength according to [17,18] were obtained and are listed with the specific specimen ages at testing in Table 2. The stress–strain relationship for the concrete used for the calculation was taken from [19] and is shown in Figure 3. Up to 40% of *f*_cm_ (27.3 MPa), linear-elastic behavior can be assumed according to [19]. This corresponds to a concrete strain of −0.79‰.

In addition to the compressive strength, also the concrete tensile strength *f*_ct_ was of great importance for the calculation and design, especially for deformations and deflections. The direct experimental determination of this material parameter is very complex and the results are not very reliable or robust [20]. It is not possible to apply the load without affecting the results by eccentricities. Therefore, test concepts and specimens, which are less prone to error, are used. To derive the mean uniaxial tensile strength *f*_ctm_ needed for calculation, splitting tensile tests on cylinders (Ø 150 mm, h = 300 mm) and flexural bending tests on prisms (40 × 40 × 160 mm^3^) were conducted. *f*_ctm_ was determined from the splitting tensile strength *f*_ctm,sp_ by Equation (1) as per Eurocode 2 [19] to be 3.9 MPa.

(1) $$f_{\mathrm{ctm}}=0.9\cdot \;f_{\mathrm{ctm},\mathrm{sp}}$$

The flexural tensile strength *f*_ct,fl,m_ can be converted using Equation (2) according to Eurocode 2 [19], yielding a value of *f*_ctm_ = 6.2 MPa. According to Model Code 1990 [21], Equation (3) gives *f*_ctm_ = 4.3 MPa, with a similar approach in Model Code 2020 [22] resulting in the same value. Alternatively, *f*_ctm_ can be calculated from the mean concrete compressive strength *f*_cm_ using Equation (4) according to [19]. The corresponding cracking strain *ε*_ct,cr_ = *f*_ctm_/*E*_cm_ was also determined. For small tensile stresses, it is suitable to use the same Young’s modulus as for compression when calculating the strain value. The results are summarized in Table 3.

(2) $$f_{\mathrm{ctm}}=\frac{f_{\mathrm{ct},\mathrm{fl},m}}{1.6-h/1000}$$

(3) $$f_{\mathrm{ctm}}=f_{\mathrm{ct},\mathrm{fl},m}\cdot \frac{1.5\;\cdot {\left(\frac{h}{100\;\mathrm{mm}}\right)}^{0.7}}{1+1.5\;\cdot {\left(\frac{h}{100\;\mathrm{mm}}\right)}^{0.7}}$$

(4) $$f_{\mathrm{ctm}}=2.12\cdot \ln\left[1+\left(f_{\mathrm{cm}}/10\right)\right]=2.12\cdot \ln\left[1+\left(\frac{63}{10}\right)\right]=4.2\;\mathrm{MPa}\;\mathrm{for}\;>\;\mathrm{C50/60}$$

with *h* = 40 mm (specimen height).

### 2.2. Material Properties of Carbon Reinforcement

#### 2.2.1. Small Scale Specimens and Test Setup

For the determination of the mechanical material properties of the CRC, small-scale specimens for tensile tests were manufactured. These can be used to obtain the uniaxial tensile strength of the carbon reinforcement, stress–strain behavior, and ultimate strain. In addition, the formation of cracks can be derived. The tests were conducted according to the Guideline for Concrete Structures with Non-Metallic Reinforcement by the German Committee for Structural Concrete (DAfStb) [23]. The guideline was not completed and published at that time but the test concept was later standardized in the guideline in the same manner as performed.

The specimens were manufactured as a rectangular concrete body reinforced with one layer of carbon grid. The dimensions were 1200 × 114 × 30 mm^3^. Since the structural system of the latter slab elements corresponds to a one-way slab with the warp yarns parallel to the decisive load-bearing direction, the warp yarns had to be tested. Each specimen was reinforced with three fiber strands over the whole length in the direction of the longitudinal axis, providing a total fiber cross-sectional area of 10.9 mm^2^. Overall, two test series consisting of six specimens each were produced. Series 1 was produced in the laboratory and the determined material properties were used for the design and therefore also for the calculations in this paper. These results are presented in the following Section 2.2.2. The second series 2 was produced with cast in situ concrete and served as a control batch. The results were similar to series 1 and no peculiarities were found.

For testing, the specimens were clamped in the testing machine using clamping devices for the loading setup. The tests were performed in a displacement-controlled manner after 27 and 28 days, respectively. A detailed description of the test concept as well as the experimental setup can be found in [24].

#### 2.2.2. Tensile Test Results

The tensile tests were carried out successfully. All specimens failed due to a rupture of the yarns combined with concrete splitting in the textile plane. The test results of series 1 (laboratory production) were very uniform and are summarized in Table 4. They displayed the high strength of the carbon reinforcement resulting in a mean value for the tensile strength *f*_f,nm,m_ regarding the fiber cross-sectional area of 3815 MPa. The statistical evaluation showed only a small scatter of the results. The characteristic value *f*_f,nm,k_ of 3383 MPa was calculated by Equation (5) with a statistical factor *k*_n_ = 1.77 (depending on the number of six specimens according to the specifications in [25]).

(5) $$f_{f,\mathrm{nm},k}=f_{f,\mathrm{nm},m}\cdot \left(1-k_{n}\;\cdot \;{v\;}_{f_{f,\mathrm{nm},m}}\right)$$

**Figure 4:**
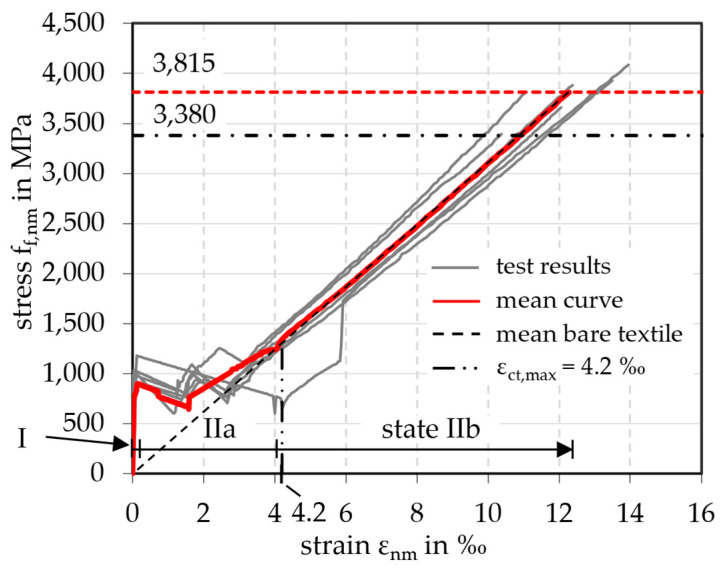
Stress–strain curves of CRC (laboratory production).

Figure 4 shows the stress–strain curves of the six specimens of series 1 as well as the mean curve (red). Also, the dashed lines give the level of the mean value of the tensile strength (red) and the characteristic value (black). The curves can be divided into three parts: uncracked state (I) up to 0,06‰, crack formation (IIa) up to 4.20‰, and cracked state (IIb). The transition from state I to state IIa happens at the appearance of the first crack and is typically characterized by a decrease in stress in the stress–strain curves due to the displacement-controlled tests. It is clearly visible in Figure 4 at approximately 900 MPa.

In state IIa, only a few cracks can be observed in which all the deformation takes place, resulting in a large drop in load. This behavior is expected to be due to shrinkage-related cracking as discussed in detail in [26]. As the concrete shrinks, the carbon fibers in the yarns are slightly compressed as they have little or no resistance to compressive stress due to their low axial stiffness in compression. When the cracks open during subsequent loading in the tests, the fibers must first be stretched again until they can absorb the tensile load. This results in a pseudo-strain of the yarns, where a part of the elongation occurs without any load being applied. The total shrinkage strain was estimated to be *ε*_cs_ = 0.33‰ after 28 days, based on calculations. Autogenous shrinkage and drying shrinkage contributed *ε*_ca_ = 0.08‰ and *ε*_cd_ = 0.25‰, respectively. The calculation methodology according to [19] is detailed in Appendix C for the full-scale slab elements presented in Section 3. For the tensile specimens, the only differing parameters were the cross-section *A*_c_ = 114 × 30 = 3420 mm^2^ and the perimeter *u* = 2 × (114 + 30) = 288 mm, resulting in a drying shrinkage strain of *ε*_cd_ = 0.29‰. The total shrinkage strain corresponds to a compression of the carbon grid of Δ*L* = 1200 mm · 0.37‰ = 0.44 mm.

As a further consequence, no tension stiffening was visible in the stress–strain curves. The mean curve fits the curve of the mean bare textile (without concrete) in state IIb. Therefore, in the calculation in Section 4, the contribution of the concrete between the cracks could not be considered on the reinforcement side. Consequently, the linear curve of the bare carbon grid was used, shown in Figure 4 as a straight dashed line from the origin to the maximum tensile strength. The mean Young’s modulus *E*_f,nm,m_ of the bare carbon grid can be determined from the slope at the state IIb of the mean curve. It was calculated to be *E*_f,nm,m_ = 310,163 MPa.

## 3. Slab Elements and Test Concept

### 3.1. Slab System in the BOX

According to German law, components of general building construction must be designed in accordance with state-of-the-art codes and standards as well as additional regulations and guidelines [27]. This also applies to unreinforced and reinforced concrete structures. These specify the calculation methods for serviceability and stability, the structural design, and material parameters. The products and materials to be used must therefore meet the requirements of these regulations. For the non-regulated, innovative materials and components in the research building CUBE, such generally applicable, normative regulations were not yet available. Therefore, individual building approvals and project-related construction-type approvals were necessary. The content of these individual building approvals includes specifications for the detailed component structure, the materials to be used including their properties, experiments, and specification of the calculation approaches.

This processing was also required for the individual approval of the building section BOX. For this, prefabricated one-way slabs were used for the intermediate floors and the roof. Typically, in steel-reinforced concrete, filigree slabs are provided with lattice girders and additional reinforcement and formed into a solid cross-section with in situ concrete. Taking advantage of the significantly higher tensile strength of carbon reinforcement (see Table 4), the required slab thicknesses are calculated to be much less than for a comparable steel-reinforced concrete slab. However, with respect to the serviceability limit state (SLS), deformations of unacceptable magnitude are to be expected. Since the explicit goal of the BOX components was to use as little material as possible, an optimization problem arose between sufficient flexural stiffness and the smallest possible concrete volume. The solution was a slab with hollow bodies, realized with rectangular blocks of OSB (oriented strand board) panels measuring 270 × 190 mm^2^. This resulted in a saving of approximately 62% of the concrete volume in comparison to a solid cross-section.

**Figure 5:**
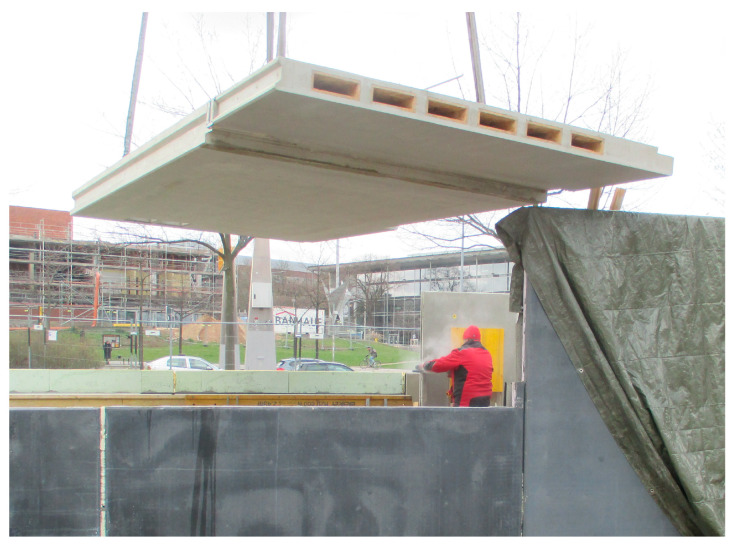
Assembly of a hollow-core slab element in the BOX. Photo: Molitor (TUD).

The CRC hollow-core slabs are approximately 4.68 m long and 2.62 m wide, with a total height of 250 mm. Some slabs were extended to 5.70 m in length with an additional balcony extension, measuring 160 mm in height and 825 mm in length. The assembly of one of those elements is shown in Figure 5. All elements have longitudinal edge beams with solid sections to join them together. The joints were cast in place to interlock the slabs. At their edges, the elements are supported by the wall elements. The production and the geometry are described in detail in the following Section 3.2.

For the structural design of the slabs and to obtain individual approval, some verifications of standard analytical calculations could be made, such as for the flexural capacity, taking into account the different properties of the carbon reinforcement. This was not possible for the verification of the shear capacity of the unreinforced webs (explained in detail in [6] and not part of this paper) and for the load distribution within a slab. Therefore, this was done experimentally with three large-scale, idealized test specimens, described in the following sections.

### 3.2. Production of the Large-Scale Test Specimens

**Figure 6:**
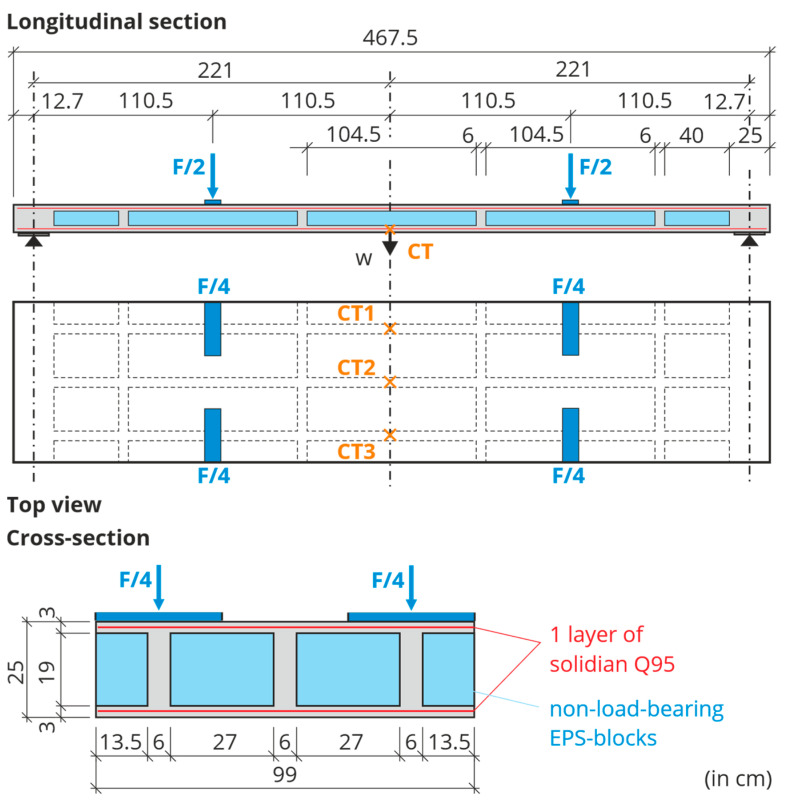
Configuration of the large-scale test specimens and test setup [6].

The slab elements for the BOX, as well as the large-scale test specimens, were produced in the same way in a prefabrication factory with a high level of quality assurance. Three test specimens were manufactured, hereinafter referred to as slab 1, slab 2, and slab 3. Their longitudinal and cross-sections are shown in Figure 6. Compared to the real slab elements in the building, the width of the specimens was reduced to 990 mm. This was sufficient for the experimental study of flexural behavior and ultimate capacity. For a realistic approach, three webs were included in the cross-section.

The concrete was poured in layers and stepwise to create the cross-section. First, the lower chord of the cross-section, with a thickness of 30 mm, was produced. The concrete was poured into the formwork and compacted until reaching a thickness of 15 mm. Then one carbon grid was inserted and covered with another 15 mm layer of concrete. Now the hollow core bodies were added on top. For the test specimens, these were not OSB elements, but expanded polystyrene (EPS) cuboids, which are less complicated to manufacture. To prevent the EPS from absorbing too much water and affecting the water-cement ratio of the concrete, it was impregnated with a release agent. The blocks had to be fixed in position horizontally at a distance of 60 mm (the thickness of the future webs) and also vertically to prevent buoyancy during concreting. The next step was to fill in the spaces between the EPS blocks and pour the first 15 mm layer of the upper chord, which had enough mass to prevent the blocks from buoyancy so that the original vertical fixation could be removed. Again, a carbon grid was inserted and covered with 15 mm of concrete. The chord thickness was controlled at random. After concreting, the specimens remained in the formwork in the enclosed production hall until transport and were protected against drying out.

### 3.3. Test Concept

**Figure 7:**
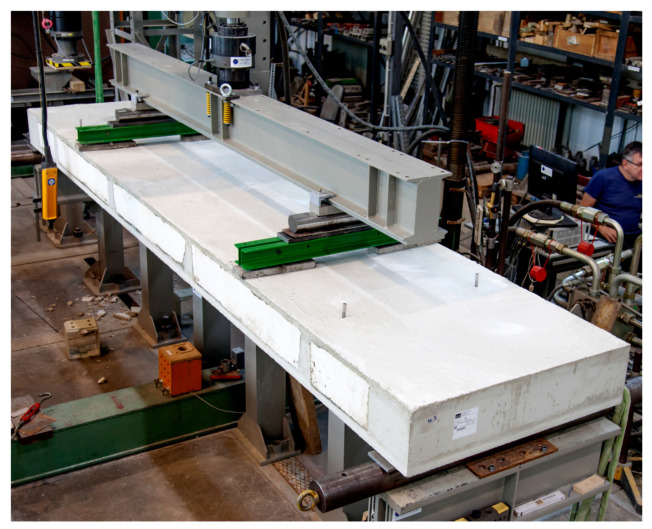
Test setup with slab specimen and loading situation [6].

The three slabs were tested at 28–31 days of age at the Otto Mohr Laboratory in Dresden, Germany. Figure 7 shows the experimental setup for the four-point bending tests. Each specimen was supported on steel rollers for free rotation. The same was done for the load application points. The load *F*/2 was applied by the testing machine at quarter points of the effective length *l*_ef_ = 4.42 m. Therefore, the ratio between the maximum bending moment and the associated maximum shear force corresponds to a distributed load. Only the two outer longitudinal webs were loaded (with *F*/4) to verify that the load would be redistributed laterally to the inner longitudinal web. The load distribution across the width was secured by steel beams. The structural system and its distances as well as the loading points are shown in Figure 6.

The moderate loading speed of 0.1 mm/s was applied by means of displacement control of the testing machine. To measure the mid-point deflection of the three longitudinal webs, three cable transducers (CT) were installed across the width, see Figure 6.

### 3.4. Slab Test Results

**Figure 8:**
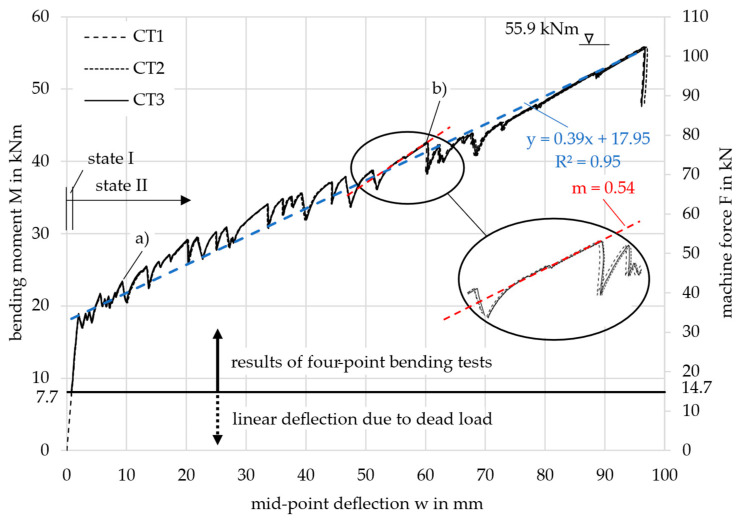
Results of the three cable transducers (CT) of slab specimen 3.

For each of the three tested slab specimens, the three cable transducers gave the same machine force over the mid-point deflection result. Exemplarily, this is shown for slab 3 in Figure 8. At an ultimate force of *F*_u_ = 101.9 kN (*M*_u_ = 55.9 kNm), the specimen failed with a deflection of *w*_u,m_ = 96.8 mm (mean value of the three cable transducers). This proves, that the inner web has been activated to contribute to the load-bearing capacity. The load was successfully redistributed by the transverse webs. The failure mode for all three slab specimens was shear failure in the area between the load introduction and the support. At the same time, only 60% of the bending moment capacity was utilized. The recalculation of the ultimate capacities is beyond the scope of this paper and is described in more detail in [6].

Noticeable in the graphs in Figure 8 is that, up to a force of approximately *F* = 80 kN, there were significant drops in load whenever a crack occurred, as also observed in the tensile test results discussed in Section 2.2.2. For instance, at approximately *w* = 9 mm (point (a)) the force dropped by 43 kN − 38 kN = 5 kN, and at around *w* = 60 mm (point (b)), the force dropped by 78 kN − 70 kN = 8 kN. The significant drop in load is likely attributable to the previously mentioned shrinkage-related cracking, which ultimately leads to reduced global bending stiffness and altered deformation behavior. After each crack and subsequent load drop, a small start-up phase with a logarithmic gradient was observed before the progression resumed in a linear manner. Due to the reduced global bending stiffness of the slab following each crack, the linear progression after each load drop exhibited a smaller gradient compared to earlier stages. In Appendix C, the calculation of the total shrinkage strain is demonstrated, leading to *ε*_cs_ = 0.33‰ and a compression of Δ*L* = 4675 mm × 0.33‰ = 1.54 mm after 28 days, according to [19].

To better illustrate this effect, a blue linear regression line was added to the *M*-*w*-curve in state II, spanning from the first crack to the ultimate load. This regression demonstrates a global gradient of *m* = 0.39. In contrast, a higher local gradient of *m* = 0.54 (red) is observed up to point (b), following a single crack occurrence. Cracks occurring prior to this point exhibit a stiffer local linear progression, as fewer cracks correspond to a higher global bending stiffness. In contrast, cracks occurring after this point result in a weaker progression. This analysis highlights that with smaller load drops, the global deformation behavior would exhibit significantly greater stiffness.

**Figure 9:**
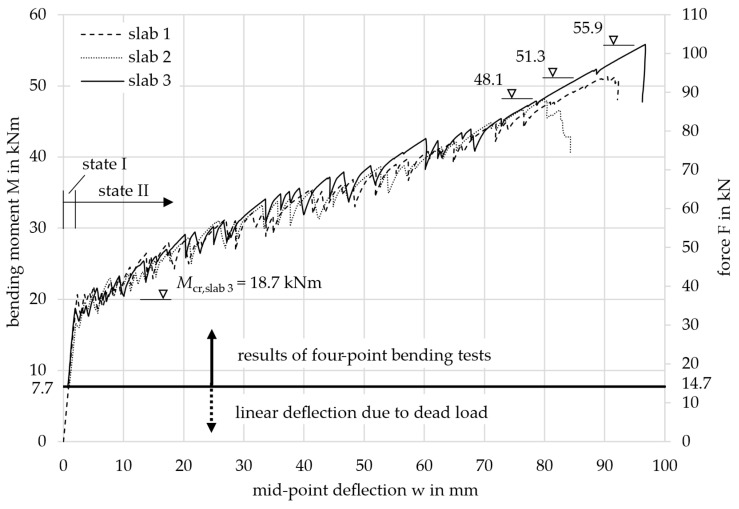
Mean load-deflection curves of the three tested slabs.

To compare the three tested slabs 1, 2, and 3, their results are displayed together in Figure 9. It shows the acting bending moment *M*, resulting from the machine force *F*, together with the mean mid-point deflection *w* measured by the three cable transducers for each slab. The bending moment was calculated taking into account both the machine force and the dead load of the specimen. When the specimens were installed in the testing machine, the dead load was already active and forced a deflection in the slab. The deflection increased linearly as the cracking moment was not yet exceeded. The influence of the dead load is indicated in the diagram by the black horizontal line and the dashed part of the curve. The dead load was composed of the concrete (1364.6 kg or 13.65 kN), the rectangular EPS blocks (17.7 kg or 0.18 kN), and the steel load setup components (82.0 kg or 0.82 kN). This resulted in a total dead load of 14.7 kN (*M* = 7.7 kNm).

All three curves showed the same two-step progression: First, the bending moment increased linearly until the first crack appeared experimentally at approximately *M*_cr,exp_ = 20.0 kNm. This initial crack formed in the region of the highest load, located at the center of the slab. Since the concrete tensile strength is statistically subject to significant scatter, the first crack likely occurred in a section where the tensile strength was lower than the mean value of the slab (*f*_ct,exp_ < *f*_ctm_).

In the second step, continuous cracking with flexural cracks until failure could be observed, leading to the oscillating curve progression. Continuous cracking occurs when, as the load increases, more and more sections of the slab towards the supports exceed the cracking moment. The moment deflection curves also showed an almost linear progression in state II due to the linear-elastic material behavior of the carbon reinforcement, see Section 2.2.2. The slabs failed at 51.3 kNm (slab 1), 48.1 kNm (slab 2), and 55.9 kNm (slab 3) due to shear between the load introduction and the supports. The failure was indicated by the large deformations of more than 80 mm.

For further considerations and comparisons on the deformation, the level of the SLS has to be defined. For the structural design of the slabs in the building, a bending moment of *M*_SLS_ = 13.7 kNm was calculated for the decisive load combination, see the calculation below. Therefore, the system was still in the uncracked state (13.7 kNm < 20.0 kNm) with very small deflections, see Figure 9. The limit value regarding deflections in the SLS was set to 17.7 mm (*l*_ef_/250).
Dead load$g_{k}=\frac{14.7\;\mathrm{kN}}{l_{\mathrm{ef}}}=\frac{14.7\;\mathrm{kN}}{4.675\;m}$= 3.1 kN/mService load*q*_k_= 2.5 kN/mLoad combination*p*_SLS_ = *g*_k_ + *q*_k_ = 3.1 + 2.5= 5.6 kN/mBending moment$M_{SLS}=\frac{p_{SLS}\cdot l_{ef}^{2}}{8}=\frac{5.6\cdot 4.42^{2}}{8}$= 13.7 kNm

## 4. Investigations on the Deflection

### 4.1. Calculation Methodology

For the design, the cross-sections of these CRC slabs were divided into a compressive zone and a tensile zone, where the latter can be idealized as a reinforced concrete member under uniaxial tensile loading. This was represented by the tensile test specimens in Section 2.2. As a result of the loading, the tensile member was subjected to cracking and deformation, resulting in cracked and uncracked concrete states along the component.

With the validity of Bernoulli’s hypothesis of flat cross-sections remaining flat, deformations due to shear can be neglected. By that the deflections *w* perpendicular to the longitudinal axis are dependent on the cross-sectional distortion or curvature *κ* as stated in Equation (6).

(6) −w=κ

By integrating the cross-sectional curvature Equation (6) two times, the deflection of the slab specimens can be calculated. This was done with an analytical solution for the moment-curvature relationship. This relationship can be divided into two states: The uncracked state I of the specimens and the cracked state II, which can also be seen in the experimental results in Section 3.4, see Figure 9. Unlike the steel-reinforced components, there was no yielding associated with a large increase in deformation.

For state I, the general rules of technical mechanics apply. The curvature κ^I^ along the component length was determined by the bending moment *M* and bending stiffness *E*_c_*I*^I^ with Equation (7) from [19]. The Young’s modulus of the concrete was determined by cylinder tests, see Section 2.1.2. For the moment of inertia *I*^I^, the parameters of the carbon reinforcement have been taken into account in a mechanically correct way with the specific ratio of the moduli of elasticity *α*_nm_ = *E*_nm_/*E*_c_. The bending stiffness was calculated to be *E*_c_*I*^I^ = 29.0 MNm^2^. Since the cross-sectional geometry, including the materials, remained the same along the length of the slab, the *M*-*κ*-relationship as well as the bending stiffness *EI*^I^ for state I was valid for the entire slab at all load steps.

(7) $$\kappa^{I}=\frac{M}{E_{c}\cdot I^{I}}$$

The transition from state I to state II happens when the cracking moment *M*_cr_ is reached. The cracking moment is highly dependent on the tensile strength of the concrete. This is subject to a large scatter not only as a material parameter itself, but also along the geometrical dimensions (length, width, height) of the component, which was expected to be even further increased by the slender cross-section. Therefore, this parameter was difficult to predict, even with experimental results. Comparisons of calculated uniaxial tensile strength values *f*_ctm_ are shown in Table 3 in Section 2.1.2. Furthermore, the transition to state II in the calculation is characterized by a significant jump in deflection, resembling the behavior of a load-controlled test. In contrast, the slab tests exhibited this transition as an inflection point, with no jump in deflection but rather a drop in load after each crack formation, due to the displacement-controlled nature of the test.

**Figure 10:**
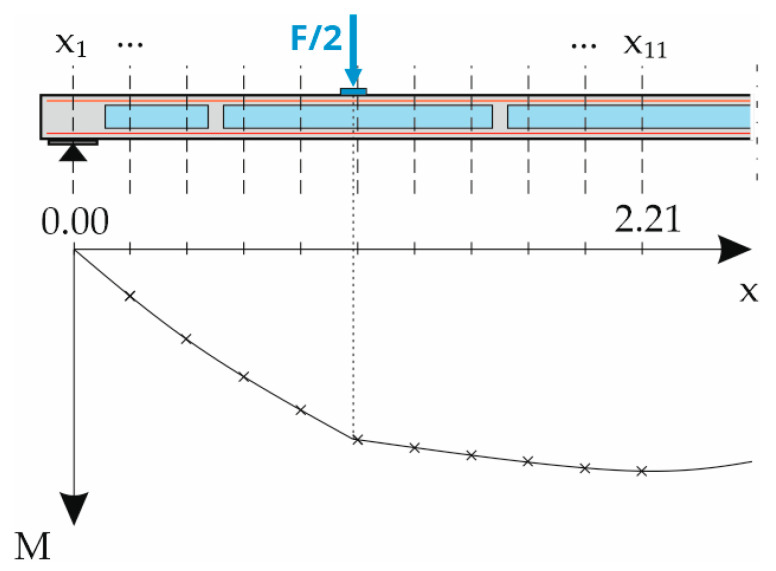
Nodes *x*_i_ along the slab element and bending moment distribution.

To calculate the deflections in states I and II, the slab was divided into 20 equidistant parts along its length, resulting in 21 nodes *x*_i_ to be calculated. For the symmetrical slab elements, it was sufficient to consider only one half of the slab and later add the deflection results for each half. Therefore, only 11 nodes from *x*_1_ to *x*_11_ had to be calculated, see Figure 10. This was done in the form of a table. Since the component tests were conducted in four-point bending tests with simple supports, the maximum bending moment at each loading state was at the center of the slab (node *x*_11_ = 2.21 m) as a combination of the element’s dead load and the machine force. Therefore, the first crack appeared in the center of the slab with the maximum bending moment equal to the cracking moment, while the rest of the slab was still in state I.

With the results of the bending moment and cross-sectional curvature for each node *x*_i_ the moment–curvature relationship was set up and numerically integrated by a Newton-Cotes-formula to obtain the mid-point deflection *w*. The Simpson’s rule uses a polynomial with three support points. The integral was calculated by Equation (8) [20]. Since linear behavior was assumed for the moment–curvature relationship in state I, only one load step for reaching the cracking moment needs to be considered.

(8) $$w=2\;\cdot \int_{x_{0}=0}^{x_{n}=\frac{l}{2}}\kappa \cdot \;\delta Mdx$$

Setting up the moment–curvature relationship in state II was more complicated since it depends on the strain distribution across the depth in the cracked cross-section. In the following, an example of the calculation will be shown with slab 3, because it had the highest ultimate failure load.

In order to reproduce the now overall nonlinear behavior of the component with increasing load, four discrete load steps (LS), the ultimate load, and three steps between the cracking moment and ultimate load were calculated in state II and linearly connected, as shown in Table 5. Load step LS 2 was chosen, when the experimental and calculated deflection values (state II) differed by less than 10 mm, as shown in the next section. LS 1 and LS 3 were chosen between the load at the first crack and LS 2, and between LS 2 and the failure load, respectively.

At each of these load steps, the component was again divided into 10 equidistant parts with 11 nodes mentioned above, see Figure 10. Each of these nodes was subjected to a different bending moment and different internal forces. It was therefore necessary to iterate the correct strain distribution across the depth for all of them until equilibrium was reached between the internal and external bending moment.

For each of the chosen load steps, LS, the external bending moment was known due to the known boundary conditions and loading situation. First, the strain distribution was estimated by assuming the tensile strain in the reinforcement and calculating the concrete strain to achieve an equilibrium of the internal forces and moments. A linear-elastic stress–strain relationship was used for the carbon textile grid, as shown in Figure 4, and the nonlinear stress–strain law was used for the concrete, as shown in Figure 3. Using the estimated strain distribution, the internal bending moment capacity was calculated from the resulting internal forces. The internal bending moment was then compared to the external bending moment: if the internal bending moment was smaller, the reinforcement strain assumed was too small and needed to be increased, and vice versa. The iteration process is shown in detail in Appendix A.

When the equilibrium of moments was found, the associated strain distribution was used for the calculation of the curvature. In addition, the height of the compression zone *x*^II^ and the inner lever arm $z_{\mathrm{nm}}^{\mathrm{II}}$ could also be obtained in state II. With the strain distribution along the depth *d*_nm_, the axial stiffness *E*_nm_·*A*_f,nm,_ and the load-dependent geometric parameters *x*^II^ and $z_{\mathrm{nm}}^{\mathrm{II}}$, the bending stiffness *EI*^II^ was calculated according to Equation (9) from [19]. At higher load levels, the compression zone was confined to the top chord. Therefore, the parameters in Equation (9) changed only slightly and the bending stiffness remained almost constant. With *E*_nm_ = 310,163 MPa, *A*_f,nm_ = 94.3 mm^2^/m, $z_{\mathrm{nm}}^{\mathrm{II}}$ = 228.8 mm, *d*_nm_ = 235 mm and *x*^II^ = 18.5 mm, *EI*^II^ becomes 1.44 MNm^2^.

(9) $${\mathrm{EI}}^{\mathrm{II}}=E_{\mathrm{nm}}\cdot \;A_{f,\mathrm{nm}}\;\cdot \;z_{\mathrm{nm}}^{\mathrm{II}}\;\cdot \;\left(d_{\mathrm{nm}}-x^{\mathrm{II}}\right)$$

In summary, a total of 33 iterations for the strain distribution across the depth were required for the considered load steps in state II, each with 11 nodes.

The curvature *κ*^II^ in state II was calculated with the carbon strain *ε*_nm,m_ by means of Equation (10) from [19].

(10) $$\kappa^{\mathrm{II}}=\frac{\varepsilon_{\mathrm{nm},m}}{d-x^{\mathrm{II}}}$$

### 4.2. Calculation Results

**Figure 11:**
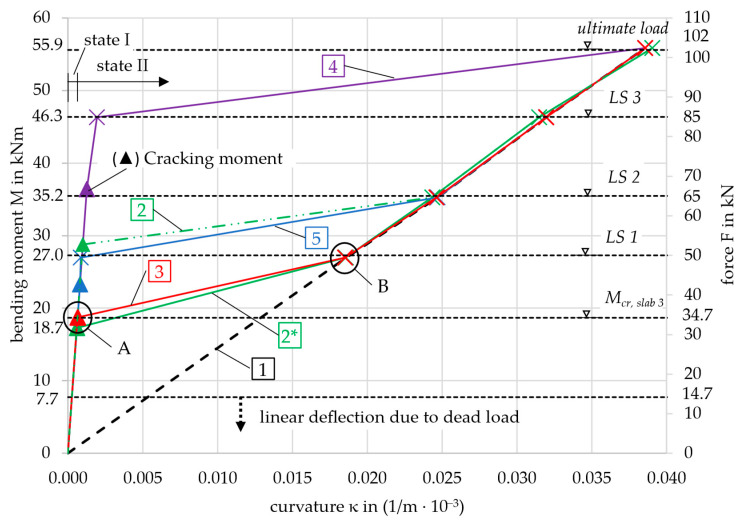
Calculated moment–curvature relationships.

With the calculation methodology known, the moment–curvature curve can be drawn. In order to represent the test results as accurately as possible, the mean values of the material properties from Table 2 and Table 4 were used. In Figure 11, several moment–curvature relationships are drawn. The differences arise from varying values of the concrete tensile strength used in the calculation, as shown in Table 3. The subsequent analysis considers the curves in Table 6:

Curve no. 6 (index 6) was calculated with *f*_ctm,6_ from Table 3.1 in [19] (concrete strength and deformation parameters) for a concrete strength class of C55/67 and a simplified approach for the deflection according to [19]. This does not require the moment–curvature relationship, which is why curve no. 6 is not included in Figure 11. It will be discussed in Section 4.3. Curves no. 2 and 2* both show the calculated *M-κ*-curves with the concrete tensile strength from prism tests according to [19]. For curve no. 2*, *f*_ctm,2*_ was modified, which will be discussed in Section 4.3.

All curves have markers where they meet the discrete load levels at which the deflection was calculated, also indicated in the figure by horizontal dashed lines. The triangles on the left side of the diagram represent the cracking moment for each curve. These values, along with their corresponding deflections, are summarized in Table 7.

Each graph that includes the effect of concrete tensile strength shows two distinct inflection points, labeled A and B. For illustration, these points are highlighted on curve 3 in Figure 11. Point A marks the transition beyond which no contribution from the concrete in the tensile zone is considered in the calculation, which will be explained in detail in Section 4.3. Consequently, point B is defined, where the calculation aligns with curve 1, representing only state II. In curve 1, starting from the origin, the cross-section is defined solely by the concrete compressive zone and the reinforcement. The curve exhibits linear behavior up to the point of failure (*M*_u_ = 55.9 kNm), characterized by strain values *ε*_c_ = −0.72‰ and *ε*_nm_ = 8.35‰, with a compressive zone height *x*^II^ = 1.85 mm. At this stage, the neutral axis lies within the top chord, and the concrete remains in a linear-elastic state, see Figure 3.

Firstly, the focus is placed on curve 2 to show the significant influence of the concrete tensile strength. The first crack was expected to appear at a calculated bending moment of *M*_cr,2_ = 28.8 kNm when exceeding the concrete tensile strength of *f*_ctm,2_ = 4.3 MPa, see Table 7 or Section 2.1.2. At loads below the cracking moment, the curvatures are very small due to the high bending stiffness in the uncracked state. When exceeding the cracking moment, the curvature increases rapidly. The bending moment for the sections at the nodes *x*_i_ was variable along the length of the slab. Depending on this, the curvature value was now assigned to each section. With the integration, the mid-point deflection of the slab was calculated.

**Figure 12:**
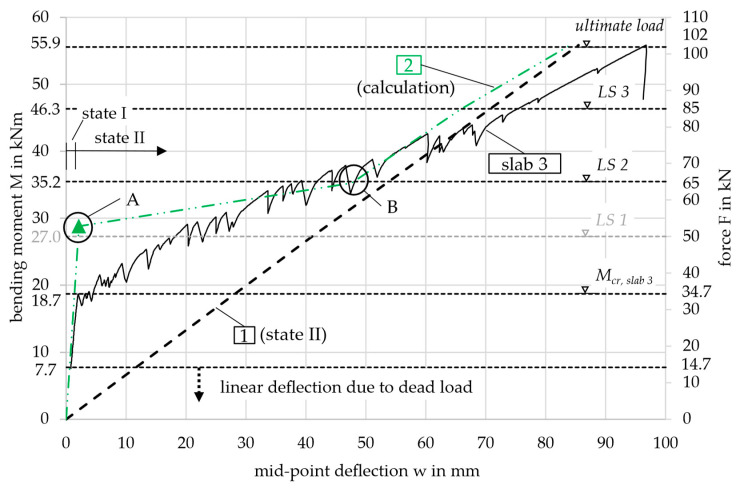
Moment-deflection diagram, comparison of experimental and calculation results.

The deflection as a function of the bending moment was then compared with the experimental results. This is shown in Figure 12 for slab 3, which had the highest ultimate capacity among the three slabs, see Figure 9. The load steps LS 1–3 and the ultimate load considered in the calculation are again indicated by the horizontal dashed lines in the following diagram. Since LS 1 is smaller than the cracking moment (27.0 < 28.8 kNm), it is not relevant for the deformation calculation of curve 2 (which exhibits linear behavior in state I). As a result, the horizontal dashed line is shaded gray.

Figure 12 shows, that the mid-point deflection of slab 3 in the 4-point bending test was underestimated by the calculation result of curve 2. In state II, with a bending moment greater than 35.2 kNm, the deformation difference was small (0–10 mm). However, since the calculated cracking moment was significantly higher than in the test, the deformation difference increased between 18.7 and 35.2 kNm. This discrepancy is due to the determination of the concrete tensile strength, which is discussed in the following section.

The inflection points A and B shown in curve 2 are not observed in the test results. This is due to the displacement-controlled nature of the test, which leads to a gradual increase in deformation and prevents sudden, large jumps.

### 4.3. Discussion on the Concrete Tensile Strength

#### 4.3.1. Simplified Approach

To compare with the complex calculation using results based on concrete tensile strengths estimated from small-scale laboratory tests, a simplified approach is presented first. The value *f*_ctm,6_ = 4.2 MPa (curve 6) was taken from Table 3.1 in Eurocode 2 [19] to calculate the cracking moment of *M*_cr,6_ = 28.3 kNm. With Equations (11) and (12) from [19] the bending stiffness *EI*^II^ = 1.44 MNm^2^ in the state II can be calculated according to Equation (9).

(11) $$x^{\mathrm{II}}=\left(-\alpha_{\mathrm{nm}}\cdot \rho_{\mathrm{nm}}+\sqrt{{\left(\alpha_{\mathrm{nm}}\cdot \rho_{\mathrm{nm}}\right)}^{2}+2\cdot \alpha_{\mathrm{nm}}\cdot \rho_{\mathrm{nm}}}\right)\cdot d_{\mathrm{nm}}=19.2\;\mathrm{mm}$$

$$\mathrm{with}\;\;\;\;\alpha_{\mathrm{nm}}=\frac{E_{\mathrm{nm}}}{E_{c}}=8.9$$

$$\;\;\;\;\;\;\;\;\;\rho_{\mathrm{nm}}=\frac{A_{f,\mathrm{nm}}}{b\cdot d}=0.0004$$

(12) $$z_{\mathrm{nm}}^{\mathrm{II}}=d_{\mathrm{nm}}\;-\frac{x^{\mathrm{II}}}{3}=228.6\;\mathrm{mm}$$

The mid-point deflections for the load contributions from the dead load and the machine load can be calculated with calculation tables for integrals for structural calculation according to Equation (13) for the distributed dead load and (14) for the loads *F*/2 from the testing machine. For the total deflection *w*_tot,_ they have to be added.

(13) $$w=\frac{5}{384}\cdot \frac{p\cdot l_{\mathrm{ef}}^{4}}{\mathrm{EI}}$$

(14) $$w=\frac{F/2\cdot l_{\mathrm{ef}}^{3}}{24\cdot \mathrm{EI}}\cdot \left(3\cdot \frac{a}{l_{\mathrm{ef}}}-4\cdot {\left(\frac{a}{l_{\mathrm{ef}}}\right)}^{3}\right)$$

The parameter *a* is the distance of *F*/2 from the support (*a* = 1.11 mm, see Figure 6).

The distributed load *p* was calculated by including the load setup components in the dead load of the slab. The overall dead load was 3.31 kN/m (14.7 kN/*l*_ef_). Two situations are presented in detail exemplarily:

1. Cracking moment (state I, *EI*^I^ = 29.0 MNm^2^)
  → Machine force *F*_cr_ = 37.4 kN (*M*_cr,6_ = 28.3 kNm), *F*_cr_/2 = 18.7 kN
  → $w_{cr,dead\;load}=\frac{5}{384}\cdot \frac{3.31\;\cdot \;4.42^{4}}{29.0}=0.6\;mm$
  → $w_{cr,machine}=\frac{18.7\;\cdot \;4.423}{24\;\cdot \;29.0}\cdot \left(3\cdot \frac{1.11}{4.42}-4\cdot {\left(\frac{1.11}{4.42}\right)}^{3}\right)=1.6\;mm$
  → $w_{cr,tot}=0.6+1.6=2.2\;mm$
2. Failure load level (state II, *EI*^II^ = 1.44 MNm^2^):
  → Machine force *F*_u_ = 87.3 kN (*M*_u_ = 55.9 kNm), *F*_u_/2 = 43.7 kN
  → $w_{u,dead\;load}=\frac{5}{384}\cdot \frac{3.31\;\cdot \;4.42^{4}}{1.44}=11.4\;mm$
  → $w_{u,machine}=\frac{43.7\;\cdot \;4.423}{24\;\cdot \;1.44}\cdot \left(3\cdot \frac{1.11}{4.42}-4\cdot {\left(\frac{1.11}{4.42}\right)}^{3}\right)=75.3\;mm$
  → $w_{u,tot}=11.4+75.3=86.7\;mm$

**Figure 13:**
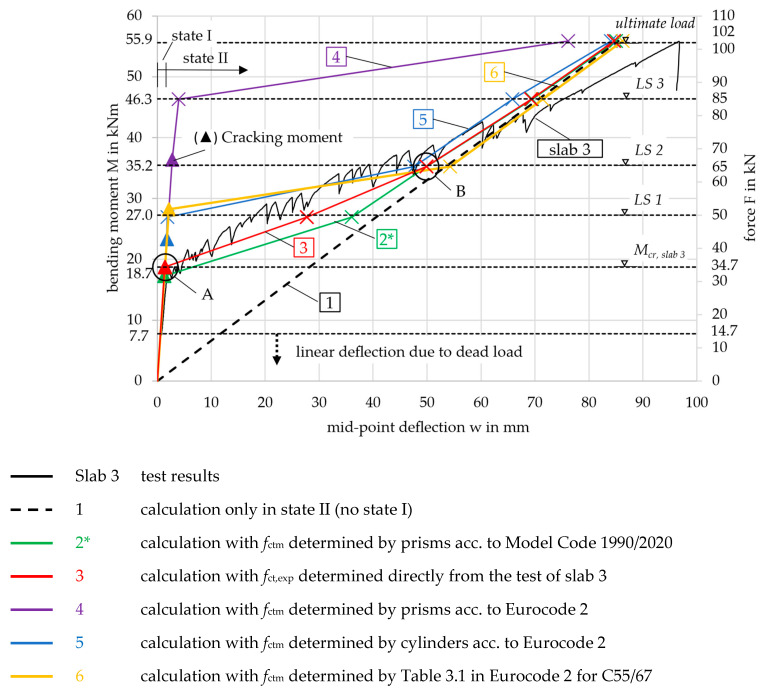
Moment-deflection diagram of experimental and calculation results for curves 1–6 (references in Table 6).

Situation 1, with a calculated mid-point-deflection of *w*_cr,tot_ = 2.2 mm, yielded the same result as the previous calculation for curve 2, as the concrete tensile strength was identical. For situation 2, the experimental deformation (w_u,exp_ = 96.8 mm) was underestimated by 10.1 mm. This discrepancy is attributed to the softer response in state II observed in the experiment, likely caused by shrinkage, as explained in Section 3.4. While the calculated results showed no significant differences, a notable gap remained between 20 and 35 kNm when comparing the calculations to the test results, see Figure 13. This will be further examined in the following chapter.

#### 4.3.2. Modification of the Concrete Tensile Strength Considering Scaling Effects

The concrete tensile strength for these slab elements was estimated from small-scale prisms (*h*_prism_ = 40 mm) produced together with the slab specimens as described in Section 2.1.2. The flexural tensile strength is subject to significant scaling effects with respect to the depth [20]. This can be taken into account by converting the flexural tensile strength of the prisms according to the height of the slab *h*_slab_ = 250 mm for a more accurate calculation result. In Equation (15) (acc.to [19]) and (16) (acc. to [21]), the conversion factors were calculated by rearranging Equations (2) and (3):

(15) $$\frac{{f\;}_{\mathrm{ct},\mathrm{fl}}^{h=250}}{{f\;}_{\mathrm{ct},\mathrm{fl}}^{h=40}}=\frac{f_{\mathrm{ctm},4}}{f_{\mathrm{ctm},4}}\cdot \frac{1.6-250/1000}{1.6-40/1000}=\frac{1.35}{1.56}=0.87$$

(16) $$\frac{{f\;}_{\mathrm{ct},\mathrm{fl}}^{h=250}}{{f\;}_{\mathrm{ct},\mathrm{fl}}^{h=40}}=\frac{f_{\mathrm{ctm},2}}{f_{\mathrm{ctm},2}}\cdot \frac{\frac{1+1.5\;\cdot {\left(\frac{250\;\mathrm{mm}}{100\;\mathrm{mm}}\right)}^{0.7}}{1.5\;\cdot {\left(\frac{250\;\mathrm{mm}}{100\;\mathrm{mm}}\right)}^{0.7}}}{\frac{1+1.5\;\cdot {\left(\frac{40\;\mathrm{mm}}{100\;\mathrm{mm}}\right)}^{0.7}}{1.5\;\cdot {\left(\frac{40\;\mathrm{mm}}{100\;\mathrm{mm}}\right)}^{0.7}}}=\frac{1.35}{2.27}=0.60$$

This resulted in flexural tensile strength for the slab specimens that was lower than the original value obtained from the prisms, along with a proportional reduction in axial tensile strength. This reduction was incorporated into the recalculation of the results from curve 2 in Section 4.2, now designated as curve 2*. The updated tensile strength value used for the calculation of curve 2* was $f_{\mathrm{ctm},2^{*}}^{*}=0.6\;\cdot \;f_{\mathrm{ctm},2}=2.6\;\mathrm{MPa}$, see Table 7. This value is closely aligned with the axial tensile strength derived from the experimental results using Equation (17) [19] and the cracking moment *M*_cr,exp_ = 18.7 kNm (see Figure 9). Table 7 also includes the adjusted value for curve 4.

(17) $$f_{\mathrm{ct},\exp}=\frac{M_{\mathrm{cr},\exp}}{W_{y}}=\frac{18.7\;\mathrm{kNm}}{0.00673\;m^{3}}=2.8\;\mathrm{MPa}$$

For curve 4, the updated tensile strength value of $f_{\mathrm{ctm},4}^{*}=0.87\;\cdot \;6.2=5.4\;\mathrm{MPa}$ was applied. The moment-curvature behavior for curve 2*, along with all the other curves, is shown in Figure 11 with a comparison of the original curve 2. The newly calculated cracking moment *M*_cr,2_^*^ = 17.3 kNm and the deformation in state I now align with the transition point A from state I to state II in the experimental curve, see Figure 13.

#### 4.3.3. Smeared Crack Model and Updated Calculation Results

**Figure 14:**
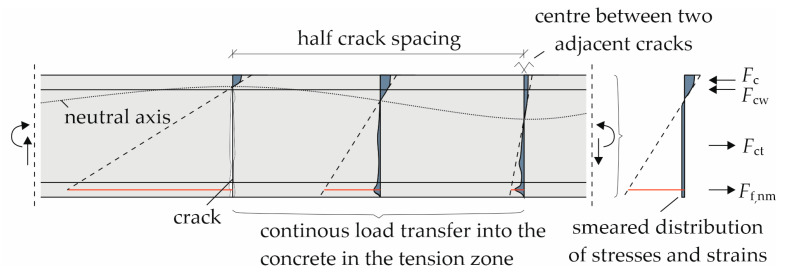
Concrete tensile strength contribution and smeared cracks from [31].

In addition to the adjustment at the material level for the concrete tensile strength value, the contribution at the cross-sectional level of the concrete in the tensile zone between the cracks was also considered here. In Section 2.2.2, it was described, that no tension stiffening effect can be considered for the material law of the reinforcement. However, the concrete below the neutral axis in between cracks acts in tension until its tensile strength is exceeded and the subsequent crack propagates towards the compression zone. This consideration of smeared cracks was introduced by *Quast* [28,29] and further developed by, e.g., *Espion* [30]. The qualitative representation is shown in Figure 14.

**Figure 15:**
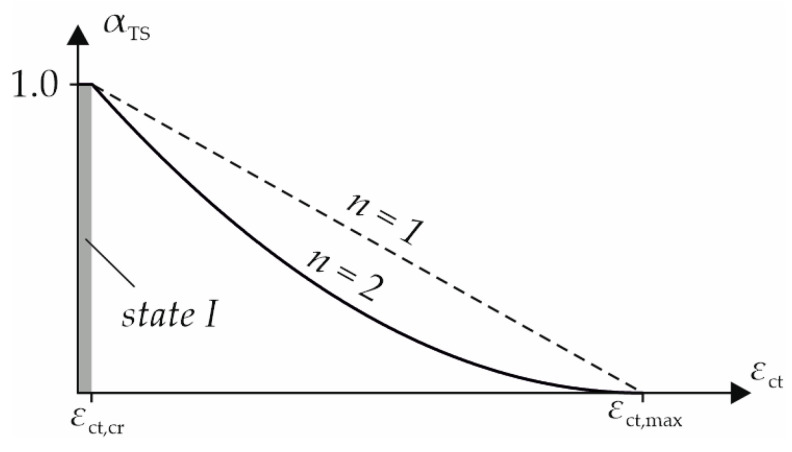
Tension stiffening reduction factor *α*_TS_ from [32].

For the contribution of the concrete to the load-bearing behavior in the tension zone, the assumption of *Quast* [28,29] has been adopted and modified for carbon-reinforced concrete. In a cross-section of steel-reinforced concrete, the concrete below the neutral axis can be fully utilized up to its tensile strength *f*_ctm_ and strain *ε*_ct,cr_. Since the tensile strength varies depending on the calculation method, the corresponding values for *ε*_ct,cr_ are presented in Table 3. Thereafter, it is reduced by the tension stiffening reduction factor *α*_TS_ shown in Figure 15 according to Equation (18) [32] until the concrete strain *ε*_ct_ reaches the yield strain of the steel reinforcement with *ε*_sy_ = *ε*_ct,max_.

(18) $$\alpha_{\mathrm{TS}}={\left(\frac{\varepsilon_{\mathrm{ct},\max}-\varepsilon_{\mathrm{ct}}}{\varepsilon_{\mathrm{ct},\max}\;-\varepsilon_{\mathrm{ct},\mathrm{cr}}}\right)}^{n}$$

The reduced concrete tensile stress *σ*_ct_ can be calculated with the reduction factor *α*_TS_ and *n* = 2 according to Equation (19).

(19) $$\sigma_{\mathrm{ct}}=\alpha_{\mathrm{TS}}\cdot f_{\mathrm{ct}}$$

As a limit for the ultimate tensile strain of the carbon-reinforced concrete, the state of the complete crack formation from the tensile tests in Figure 4 was taken as *ε*_ct,max_ = 4.2‰. Since the real concrete tensile stress distribution in each cross-section along the slab is nonlinear, the contribution is simplified in a smeared approach as a constant mean tensile stress below the neutral axis as shown on the right in Figure 14.

Here, the model has been implemented in the iteration of the correct strain distribution to obtain the reinforcement strain as explained in Section 4.1. Once the cracking moment has been exceeded, three forces act on the section now, see Figure 14: the concrete compressive force *F*_c_, the tensile force of the reinforcement *F*_f,nm,_ and the tensile force of the concrete *F*_ct_. If the compressive zone extends to the webs, a fourth component for the concrete compressive force in the webs *F*_cw_ was considered.

As described in Section 4.1, the equilibrium of forces and moments was established, determining the strain distribution of each load step considered. Given the complexity of this iteration, the freeware section editor software INCA2 (version 3.00) was used. Due to the contribution of the concrete in tension at low load levels, the carbon strains and stresses were notably reduced. This ultimately led to significantly smaller deflections compared to those calculated in Section 4.2, particularly at the moderate load step LS 1.

The modified calculation for curves 2*, 3, 5, and 6 gave a much better fit to the experimental results, see Figure 13. The cracking moments (indicated by the triangles) of curves 2* and 5 only showed small deviations from *M*_cr,exp_ = 18.7 kNm. As stated in Section 3.4, the first crack in the test occurred at the area of the highest load in the center of the slab, in a section where the tensile strength *f*_ct,exp_ determined from *M*_cr,exp_ is expected to be lower than *f*_ctm_. Therefore, it is plausible that *M*_cr,5_ = 23.3 kNm, determined using the mean value *f*_ctm,5_, is slightly higher. Following this logic, *M*_cr,2*_ = 17.3 kNm is estimated to be too small. In contrast, curves 6 and especially 4 significantly overestimated the cracking moment. *M*_cr,6_ = 28.3 kNm was not determined on small-scale specimens of the slab but rather from a design table in [19]. Curve 4 was based on the tensile strength derived from prisms using Equation (2), the applicability of which needs to be reconsidered due to the considerable difference from the experimental curve.

It is also notable that, immediately after exceeding the cracking moment, curves 2*, 3, and 6 behaved differently compared to curves 4 and 5. Due to the small estimated tensile strength, the contribution of the concrete in the tension zone was minimal with increasing load. In the simplified approach for curve 6, the tensile strength was not accounted for in the calculation formulas. The graphs 2*, 3, and 6 converged toward line 1 after exceeding the cracking moment. Curves 4 and 5, however, exhibited stiffer behavior up to the next load step (LS 1 and LS 3, respectively). Mathematically, the slab remained uncracked over large regions from the supports to the center, where the curvature of the cracked sections was kept small due to the smeared crack model.

From LS 2 onward, all graphs except for curve 4 followed curve 1, deviating only slightly, as the cross-sections closest to the supports remained uncracked and exhibited a stiffer behavior. Nevertheless, curves 2*, 3, 5, and 6 displayed a steeper gradient than the actual slab in the test, causing them to intersect the test result graph. The displacement control of the testing machine prevented significant jumps in the deflection and inflections in the graph from the cracking moment to LS 1. Furthermore, the large drops in load following each crack in the slab test resulted in a reduced global bending stiffness as explained in Section 3.4. The gradient of graph 1 in Figure 13, *m* = 0.64, representing sole state I, is more comparable to the local gradients after each crack formation—e.g., *m* = 0.54 (red in Figure 8)—than to the global bending stiffness, *m* = 0.39 (blue in Figure 8).

Overall, curves 2* and 3 yielded the best results, showing only minimal deviations from slab 3. Compared to Curves 5 and 6, the predicted deflection was on the safe side, being larger than the deflection observed in the test under moderate load levels, such as those expected under service loads. Curve 4, on the other hand, substantially underestimates the deformation, leading to the conclusion that Equation (2) from [19] is not suitable for the slab element considered in this study. The deflection at *M*_SLS_ = 13.7 kNm in the calculated curves remained well within the SLS deflection limit of *w*_SLS_ = 17.7 mm (*l*_ef_/250) for all curves.

It can be concluded that the mechanical approaches for calculating mechanical properties, stresses, strain distributions, flexural strength, and deformations adopted from steel-reinforced concrete are still valid for other composite materials such as carbon-reinforced concrete. For many of these, uniaxial tensile strength is an essential parameter that must be determined very accurately. This is even more important for the typically slender cross-sections of CRC members.

## 5. Conclusions

This paper presents the experimental investigation of CRC hollow-core slabs and the recalculation of their deflection behavior achieved in the tests. Furthermore, their behavior in load-bearing capacity was analyzed. The extensive study of these elements was necessary to get them approved for use in the world’s first building with concrete components reinforced only with non-metallic rebars and grids, called CUBE (Dresden, Germany).

The main conclusions are stated as follows:

- The production of CRC slab elements for the CUBE and three slab specimens with very slender cross-sections and hollow-core bodies was successfully completed in a prefabrication factory. The many individual steps required were carried out with great care and attention to quality assurance. The correct positioning of the reinforcement in the chords of the cross-section was of particular importance.
- The Ultimate Limit State (ULS) design in bending was adapted from conventional steel-reinforced concrete and successfully applied using the new material parameters of the carbon reinforcement. Small-scale tests provided valuable input values for accurately predicting the load-bearing behavior.
- In addition to the Ultimate Limit State (ULS), the design of the slabs in the Serviceability Limit State (SLS) was also of significant importance. Specifically, the deformation of the slabs needed to be investigated to demonstrate their minimal deflections and to provide an early indication of failure. In the tests, only small deflections were observed at moderate load levels. Up to the SLS, the specimens remained uncracked. A considerable distance to the ultimate load remained, with large deflections of over 80.0 mm and a high number of cracks indicating impending failure.
- The detailed calculation approach for accurately determining the deflections of CRC elements was also adapted from steel-reinforced concrete. The overall results were in good agreement with the experimental tests and also on the safe side for moderate loads. As presented, simplified approaches could also be adopted and modified from reinforced concrete.
- The effects of shrinkage and concrete tensile forces must be carefully considered to ensure accurate deflection calculations. In particular, the determination of tensile strength is crucial, as it significantly impacts the results at moderate load levels. The selected approach must ensure that the deflection calculations remain on the safe side. For the presented slab elements, the estimation of tensile strength from prisms using approaches from Model Code 1990 and 2020 (curve 2*), as well as from cylinders using methods from Eurocode 2 (curve 5), yielded good results. The computational method using tabulated parameters from Eurocode 2 (curve 6) also provided a good approximation of the tests, considering that the experimental value *f*_ct,exp_ is lower than the slab’s mean tensile strength. The approach with prisms in Eurocode 2 (curve 4), however, does not seem suitable for estimating the tensile strength of thin carbon-reinforced concrete components or for calculating their deflection. It significantly overestimates the cracking moment, leading to an underestimation of deformations.
- Shrinkage led to noticeable load drops in displacement-controlled tests and resulted in weaker bending stiffness than initially estimated in the calculations, particularly at higher load levels. These effects could be even more pronounced in very slender structures and cross-sections, necessitating further investigation.

## Figures and Tables

**Table 1:** Geometric properties of the carbon textile grid Q95/95-CCE-38-E2.

Test Series	Unit	Value
Fiber cross-sectional area of fiber strand	mm^2^	3.62
Fiber cross-sectional area	mm^2^/m	95.3
Grid width in warp and weft direction	mm	38
Fiber strands per Meter	-	26.3

**Table 2:** Material properties of the concrete C55/67.

Test Series ^1^	Unit	Value	*σ*	CoV	Age
Mean concrete compressive strength *f*_cm_ ^2,a^	MPa	68.3	0.93	0.01	31
Mean splitting tensile strength *f*_ctm,sp_ ^2,a^	MPa	3.9	0.24	0.06	34
Mean flexural tensile strength *f*_ct,fl,m_ ^3,b^	MPa	9.7	0.11	0.01	28
Mean Young’s modulus *E*_cm_ ^2,a^	MPa	34.733	901.9	0.03	31

^1^ 3 tested specimens for each series; ^2^ specimen storage like slab elements; ^3^ water storage (1–2 d in formwork, water storage until testing at 21 °C); ^a^ cylinder, Ø 150 mm, h = 300 mm; ^b^ prism, 40 × 40 × 160 mm^3^.

**Table 3:** Calculated mean uniaxial concrete tensile strength and strain values.

Conversion	*f*_ctm_ in MPa	*ε*_ct,cr_ in ‰
From *f*_ctm,sp_ by Equation (1) acc. to Eurocode 2 [19]	3.5	0.10
From *f*_ct,fl,m_ by Equation (2) acc. to Eurocode 2 [19]	6.2	0.18
From *f*_ct,fl,m_ by Equation (3) acc. to Model Code 1990/2020 [21,22]	4.3	0.12
From Table 3.1 in Eurocode 2 [19]	4.2	0.12

**Table 4:** Material properties of the textile grid determined according to [23].

Mean tensile strength regarding *A*_f,nm_	*f*_f,nm,m_	3815 MPa
Ultimate strain	*ε*_nm_	12.3‰
Coefficient of variation	${v\;}_{f_{f,\mathrm{nm},m}}$	0.064
Characteristic value	$f_{f,\mathrm{nm},k}$	3383 MPa

**Table 5:** Load steps for the calculation.

Load Steps	Force in kN	Moment in kNm
LS 1	50.0	27.0
LS 2	65.0	35.2
LS 3	85.0	46.3
Ultimate failure load	101.9	55.9

**Table 6:** Description of the calculation curves.

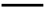	slab 3	test results
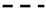	1	calculation only in state II (no state I)
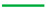	2/2*	calculation with *f*_ctm_ determined by prisms acc. to [21,22]
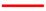	3	calculation with *f*_ct,exp_ determined directly from the test of slab 3
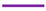	4	calculation with *f*_ctm_ determined by prisms acc. to [19]
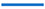	5	calculation with *f*_ctm_ determined by cylinders acc. to [19]
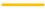	6	calculation with *f*_ctm_ determined by Table 3.1 in [19] for C55/67

**Table 7:** Comparison of the cracking moments.

		*f*_ctm_ in MPa	*M*_cr_ in kNm	*w*_cr_ in mm
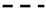	1	-	-	-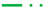	2	4.3	28.8	2.2
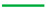	2*	2.6	17.3	1.3
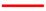	3	2.8	18.7	1.4
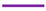	4	(6.2) ^1^ 5.4	(41.8) ^1^ 36.4	2.8
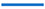	5	3.5	23.3	1.7
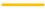	6	4.2	28.3	2.2

^1^ (*f*_ctm_) without modification (Section 4.3.2) and not used in calculation.

**Figure A1:**
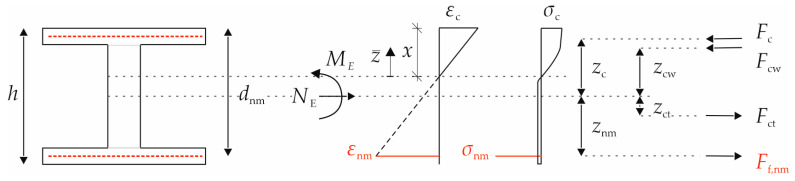
Strains, stresses, internal and external forces in a section.

## Data Availability

The original contributions presented in this study are included in the article. Further inquiries can be directed to the corresponding author.

## Appendix A. Algorithm for Iteration: Formulas According to [19]

1. Assumption of carbon strain *ε*_nm_ (positive):	$\varepsilon_{nm}$
2. Assumption of concrete strain *ε*_c_ (negative):	$\varepsilon_{c}$
3. Height of compressive zone *x*:	$x=\frac{\varepsilon_{c}\cdot d_{nm}}{\varepsilon_{c}-\varepsilon_{nm}}$
4. Resulting compressive force *F*_c_: (compression zone only in top chord)	$F_{c}=\int_{\bar{z}=neutral\;axis}^{\bar{z}=x}\sigma_{c}\left(\bar{z}\right)\cdot b\;d\bar{z}$ $\bar{z}$: Starting from the neutral axis, upward direction (see Figure A1). → Only very small concrete compressive strains of $\varepsilon_{c}<-0.63‰$ were reached. Therefore, $\bar{z}$ also a simplified approach for the compressive force on the linear-elastic branch of the concrete material law (Figure 3) can be used: $\bar{z}$ $F_{c}=x\cdot b\cdot f_{cm}\cdot 0.5$
5. Compressive force distance *a* from top edge:	$a=x-\frac{1}{F_{c}}\cdot \int_{\bar{z}=neutral\;axis}^{\bar{z}=x}\sigma_{c}\left(\bar{z}\right)\cdot b\cdot \bar{z}\;d\bar{z}$ → Simplified approach: $a=\frac{1}{3}\cdot x$
6. Resulting tensile force *F*_f,nm_:	$F_{f,nm}=\sigma_{f,nm}\cdot A_{f,nm,tot}$
7. Internal lever arms *z*_c_ and *z*_nm_:	$z_{c}=\frac{h}{2}-a$ $z_{nm}=\frac{h}{2}-d_{1}$
8. Equilibrium of horizontal forces:	$F_{c}=F_{f,nm}$
9. Equilibrium of moments:	$M_{external}=F_{c}\cdot z_{c}+F_{nm}\cdot z_{nm}$
If the moment equilibrium is reached, the iteration ends and the correct strain distribution has been determined. If the calculated bending moment from the internal forces does not match the external bending moment, the iteration must be repeated starting from step 1 with the strains adjusted.	

## Appendix B. Integration of Moment–Curvature Relationship

1. Define material properties and material laws to be used in calculation.
2. Segmentation of the construction component in equidistant points.
3. Define load steps to be considered for recalculating the moment–deflection curve (definition of calculation accuracy).
4. Calculate the bending moment along the component length (at each defined point for each load level).
5. Determine strain distribution according to Appendix A (at each defined point for each load level).
6. Calculate virtual bending moment according to virtual work method (at each defined point for each load level)
7. Deflection in state I (a) Mid-point of the component must just reach the cracking moment (for the slabs considered here). Calculate the bending moment (for each defined point along the component length) according to the machine force (in addition to the dead load) that leads to the cracking moment. (b) Calculate the curvature *κ*^I^ in state I. (c) Determining factor *f* for the numerical integration according to Simpson’s rule. (d) Integration of *M*-*κ*-relationship for deflection: $w=\sum \kappa_{i}^{I}\cdot \delta M_{i}\cdot f_{i}\cdot \frac{\Delta x}{3}$
8. Deflection in state II (a) Calculate curvatures *κ*^I^ and *κ*^II^ for states I and II. *κ*^I^ is used until reaching the cracking moment in the equidistant parts or in the defined points. When the cracking moment is exceeded, *κ*^II^ is used. (b) Integration of *M*-*κ*-relationship for deflection: $w=\sum \kappa_{i}^{I/II}\cdot \delta M_{i}\cdot f_{i}\cdot \frac{\Delta x}{3}$
9. Connect the chosen load steps (bending moment *M*_i_) in relation to the calculated mid-point deflection *w* linearly.

## Appendix C. Calculation of the Shrinkage Strain of Slab 3 According to [19]

- Total shrinkage strain: $\varepsilon_{cs}\left(t,t_{s}\right)=\varepsilon_{ca}\left(t\right)+\varepsilon_{cd}\left(t,t_{s}\right)$				(A1)
$↔\varepsilon_{cs}\left(t,t_{s}\right)=0.08+0.25=0.33‰$ , with *t* = 28 d (concrete age) and *t*_s_ = 1 d (concrete age at the beginning of drying)				
	- Basic/autogenous shrinkage: $\varepsilon_{ca}\left(t\right)=\varepsilon_{ca}\left(\infty \right)\cdot \beta_{as}\left(t\right)$			(A2)
	$↔\varepsilon_{ca}\left(t\right)=0.126‰\cdot 0.65=0.08‰$			
		- Basic notional shrinkage coefficient: $\varepsilon_{ca}\left(\infty \right)=2.5\cdot \left(f_{ck}-10\right)\cdot 10^{-6}$		(A3)
		$↔\varepsilon_{ca}\left(\infty \right)=2.5\cdot \left(60.3-10\right)\cdot 10^{-6}=0.126‰$ , with $f_{ck}=68.3-8=60.3\mathrm{MPa}$ acc. to [19]		
		- Time function: $\beta_{as}\left(t\right)=1-e^{-0.2\cdot \sqrt{t}}$		(A4)
		$↔\beta_{as}\left(t\right)=1-e^{-0.2\cdot \sqrt{28}}=0.65$		
	- Drying shrinkage: $\varepsilon_{cd}\left(t,t_{s}\right)=\varepsilon_{cd,0}\cdot k_{h}\cdot \beta_{ds}\left(t-t_{s}\right)$			(A5)
	$↔\varepsilon_{cd}\left(t,t_{s}\right)=0.34\cdot 1.0\cdot 0.74=0.25‰$			
		- Notional drying shrinkage coefficient: $\varepsilon_{cd,0}=0.85\cdot \left(220+110\cdot \alpha_{ds1}\right)\cdot e^{-\alpha_{ds2}\;\cdot \;f_{cm}/f_{cm0}}\cdot 10^{-6}\cdot \beta_{RH}$		(A6)
		$↔\varepsilon_{cd,0}=0.85\cdot \left(220+110\cdot 4.0\right)\cdot e^{-0.12\;\cdot \frac{68.3}{10}}\cdot 10^{-6}\cdot 1.36=0.34‰$		
			- Cement coefficients for CEM I, 52.5 N: $\alpha_{ds1}=4$ $\alpha_{ds2}=0.12$	
			- Relative humidity coefficient: $\beta_{RH}=1.55\cdot \left(1-{\left(\frac{RH}{RH_{0}}\right)}^{3}\right)$	(A7)
			$↔\beta_{RH}=1.55\cdot \left(1-{\left(\frac{50}{100}\right)}^{3}\right)=1.36$ , with assumed relative humidity *RH* = 50%	
		- Coefficient for notional member size: $k_{h}=1.0$ , with the notional size of the member $h_{0}=\frac{2\;\cdot \;A_{c}}{u_{c}}=\frac{2\;\cdot \;93600}{4860}=38.5\;\mathrm{mm}$		(A8)
			- Concrete cross-section: $A_{c}=2\cdot 990\cdot 30+3\cdot 60\cdot 190=93600.0{\mathrm{cm}}^{2}$	(A9)
			- Perimeter of the member: $u_{c}=2\cdot \left(990+2\cdot 30+2\cdot 135+2\cdot 270\right)+190\cdot 6=4860\mathrm{mm}$	(A10)
		- Coefficient for time development: $\beta_{ds}\left(t-t_{s}\right)=\frac{t-t_{s}}{0.04\cdot h_{0}^{1.5}+\left(t-t_{s}\right)}$ $↔\beta_{ds}\left(t-t_{s}\right)=\frac{28-1}{0.04\cdot 38.5^{1.5}+\left(28-1\right)}=0.74$		(A11)
